# Carbon Quantum Dots/Cu_2_O Photocatalyst for Room Temperature Selective Oxidation of Benzyl Alcohol

**DOI:** 10.3390/nano14020212

**Published:** 2024-01-18

**Authors:** Zhuang Tong, Yunliang Liu, Xin Wu, Yuanyuan Cheng, Jingwen Yu, Xinyue Zhang, Naiyun Liu, Xiang Liu, Haitao Li

**Affiliations:** 1 Institute for Energy Research, School of Chemistry and Chemical Engineering, Jiangsu University, Zhenjiang 212013, China; tong_zhuang@126.com (Z.T.); liuyl2024@126.com (Y.L.);; 2 Institute of Medicine & Chemical Engineering, Zhenjiang College, Zhenjiang 212028, China; liuxiang0222@126.com

**Keywords:** carbon quantum dots, photocatalysis, selective oxidation, benzyl alcohol

## Abstract

The luminescence properties and excellent carrier transfer ability of carbon quantum dots (CQDs) have attracted much attention in the field of photocatalysis. In this work, we loaded the CQDs on the surface of Cu_2_O to enhance the visible-light property of Cu_2_O. Furthermore, the composite was used for selective oxidation of benzyl alcohol to benzaldehyde. The composite catalyst achieved high selectivity (90%) for benzaldehyde at room temperature, leveraging its visible-light-induced electron transfer properties and its photocatalytic activity for hydrogen peroxide decomposition. ·OH was shown to be the main reactive oxygen species in the selective oxidation reaction of benzyl alcohol. The formation of heterostructures of CQDs/Cu_2_O promoted charge carrier separation and provided a fast channel for photoinduced electron transfer. This novel material exhibited enhanced levels of activity and stability for selective oxidation of benzyl alcohol. Potential applications of carbon quantum dot composites in conventional alcohol oxidation reactions are shown.

## 1. Introduction

The selective oxidation of alcohols to aldehydes has an important place in the fine chemical industry [1] and is also an important branch of organic synthesis. Benzaldehyde is one of the most widely used aromatic aldehydes, and can be used as a spice and flavoring agent; also, its derivatives have a wide range of applications in the field of dyestuffs, cosmetics, pharmaceutical intermediates, etc. [2]. Conventional procedures for carrying out this conversion typically rely on employing stoichiometric quantities of potent oxidizing agents (e.g., chromate, permanganate, and high-valent iodine as the inorganic oxidants) [3]. Chen [4] and Saffari’s [5] research groups obtained considerable benzaldehyde yields at high temperatures by employing noble metals Au and Pd, respectively. However, these methods have significant disadvantages, including high expenses, low productivity, stringent or fragile reaction conditions, and the generation of substantial waste by-products [6]. Besides this, conventional organic processes pose a serious threat to the development of human society. Hence, the search for novel, economical, and eco-friendly synthetic techniques for alcohol oxidation to tackle the aforementioned problems continues to be significant and captivating.

Photocatalytic oxidation technology utilizes inexhaustible solar energy, offers mild reaction conditions, produces no secondary pollution, and ensures stability and recyclability [7]. It has great potential to solve the problems of energy consumption and environmental pollution [8]. Dai et al. [9] successfully performed the selective oxidation of primary alcohols to aldehydes through photocatalysis at room temperature, utilizing O_2_ as a substitute for stoichiometric chemical oxidants (for example, Mn^VII^, Cr^VI^, and Os^IV^). Ho et al. [10] achieved 49% benzaldehyde yield by constructing a Zn_0.2_Cd_0.2_S catalyst on MoS_2_ nanoflowers, and demonstrated that the MoS_2_/Zn_0.5_Cd_0.5_S heterostructure promoted the separation of photogenerated electron–hole pairs and showed improved catalytic activities in the oxidation reaction of benzaldehyde. Wu et al. [11] synthesized ZnTi-LDH nanosheets as a photocatalyst for the selective oxidation of benzyl alcohol to benzaldehyde under visible-light irradiation and illustrated that the surface -OH groups provide additional active sites for O_2_ activation. Zhang et al. [12] synthesized InVO_4_/TiO_2_ heterojunction composite catalyst, which exhibited favorable conversion rates and selectivity towards benzaldehyde. The outstanding catalytic activity of the InVO_4_/TiO_2_ photocatalyst is attributed to the successful assembly of the heterojunction structure, promoting efficient charge separation and transfer between the components.

Carbon quantum dot (CQDs) is a new type of nanosized catalytic semiconductor with excellent photoelectric properties [13,14,15]. CQDs have gained significant attention in recent years due to their simple synthesis protocol, low toxicity, cheap cost, abundant surface functional groups, and the ability to act as electron donors or acceptors, which has made them very suitable for photocatalytic organic processes [16]. Kang et al. [17] reported the photocatalytic activity of near-infrared light-controlled CQDs for highly selective alcohol oxidation, but only carbon quantum dots are not conducive to the isolation and recycling after the reaction. Mohammadi et al. [18] obtained a non-homogeneous nanocatalyst via ionic liquid-modified carbon-quantum-dot-anchored tungstate ions (WO_4_^2−^) for selective oxidation of alcohols in the water, which showed a more satisfactory performance. However, the reaction still required heating conditions and the tedious extraction step for catalyst recycling. Problems such as low yield and the difficulty associated with carbon dot materials’ recycling have limited their application in photocatalytic organic reactions, and suitable carriers or scaffolds are required for their modification.

According to previous reports, the upconversion photoluminescence property of CQDs can effectively extend the light absorption range into the visible and even the near-infrared region [19]. Meanwhile, the carbon dots can serve as excellent electron donors, which can effectively separate photogenerated charge carriers and provide the necessary redox environment for the reaction [14]. Therefore, the organic reaction efficiency can be further improved through modifications in CQDs. CQDs have been used in conjunction with other substances to enhance the photocatalytic efficiency of reaction by exploiting various interfacial regions, increasing the abundance of charge carriers accessible for photoreaction, and facilitating charge separation. Li et al. [20] prepared Fe_3_O_4_@CdS@CQDs ternary core–shell heterostructured by in situ doping carbon quantum dots and CdS on Fe_3_O_4_ nanospheres, and used these as photocatalysts for selective alcohol oxidation. The modified CQDs act as charge mediators to accelerate the photogenerated electron–hole separation and provide active sites to facilitate the reaction. Kang et al. reported that the due to existence of CQDs in the composite, the fast electron transfer process can become faster, and the charge transfer efficiency can be enhanced [21]. The Cu_2_O can be synthesized using a simple raw material and has favorable characteristics such as an appropriate band gap, and an adjustable morphology, making it suitable for photocatalytic substrate material [22,23]. Furthermore, it is necessary to evaluate the stability of catalysts under reaction conditions since the structural morphology of the catalyst plays a major role in the selectivity of the product during the alcohol oxidation process. CQDs-based composite materials provide an effective alternative to address the selectivity and stability issues of the alcohol oxidation process [24].

In this work, a composite photocatalyst with a heterogeneous interface was prepared by combining Cu_2_O and carbon quantum dots through a facile synthesis method, and used for room temperature selective oxidation of benzyl alcohol. The microscopic morphology and structural features of the sample were studied via scanning and transmission electron microscopy, XRD, FT-IR, and Raman spectroscopy. The photo-response ability of the composites was observed via UV-Vis diffuse reflectance spectroscopy. The charge separation and movement in CQDs/Cu_2_O were evaluated via transient photocurrent response, electrochemical impedance, Mott–Schottky diagram, linear scanning voltammetry, and photoluminescence. Finally, the mechanism for the enhanced photocatalytic activity of CQDs/Cu_2_O was proposed to provide an environmentally friendly way to solve the selectivity and efficiency issues of alcohol oxidation.

## 2. Materials and Methods

### 2.1. Materials

All reagents and materials were used without further purification. Benzyl alcohol, toluene, anhydrous magnesium sulfate, copper sulfate pentahydrate, dextrose, polyvinylpyrrolidone (PVP, K30), sodium hydroxide, hydrochloric acid, and anhydrous ethanol were supplied by Sinopharm Chemical Reagent Co., Ltd. (Shanghai, China). Nitrobenzene was purchased from Shanghai Aladdin Biochemical Technology Co., Ltd. (Shanghai, China).

### 2.2. Preparation of CQDs

First, 50 mL of NaOH (1 M) solution was added to 50 mL of glucose (1 M) solution. Then, the above-mixed solution was subjected to ultrasonication (100 W) for 1 h. After the preliminary treatment, the pH of the solution was adjusted to neutral with HCl (0.1 M), following the dialyzing procedure using a semi-permeable membrane (100–500 Da) to remove impurities other than the sample of CQDs. Finally, the brownish-yellow solution of CQDs was obtained after filtration treatment with a PTFE membrane (0.22 μm).

### 2.3. Preparation of CQDs/Cu_2_O

CQDs/Cu_2_O composite material was synthesized using a one-step sonication method [25]. In a typical synthesis process, 150 mL of NaOH (1 M) solution was slowly added to 150 mL of CuSO_4_ (0.1 M) solution to form a light blue suspension. The suspension was subjected to ultrasonication (100 W) for 15 min, followed by the addition of 20 mL of poly(vinylpyrrolidone) (50 g·L^−1^) solution. Then, 100 mL of glucose (1 M) solution was slowly added to the above mixture under stirring, and then the ultrasonication was continued for 60 min again. After aging the above sample for 16 h, the crude product obtained was washed with deionized water and ethanol three times, respectively. Finally, the sample was dried in a vacuum oven at 60 °C to obtain CQDs/Cu_2_O composite with protruding structures.

### 2.4. Characterization

The prepared sample of CQDs/Cu_2_O composite was characterized via XRD, FT-IR, Raman spectroscopy, SEM, and TEM. Powder X-ray diffraction (XRD) was carried out on a XRD-6100 (SHIMADZU, Kyoto, Japan) diffractometer using 40 kV and 30 mA Cu Kα radiation at a scanning rate of 7°·min^−1^. Fourier-transform infrared spectroscopy (FT-IR) was recorded in the range of 4000–400 cm^−1^ on an iS50 FT-IR (Thermo Fisher Scientific Inc., MA, USA) spectrometer using a DTGS KBr detector. Raman spectroscopy (Raman) was performed using a confocal Raman system (RTS2, Zolix, Beijing, China) with an excitation source of 532 nm. Transmission electron microscopy (TEM)(JEM-2010, JEOL Ltd, Tokyo, Japan) and high-resolution transmission electron microscopy (HRTEM) were used to observe the morphology of the material. Gas chromatography (GC) was performed using an Agilent 6890 N (Agilent Technologies, Inc., CA, USA) with a hydrogen flame ionization detector and a 30 m Agilent polysiloxane HP-5 column (0.32 mm ID, 0.25 µm) to analyze the product formation. Nitrogen was used as the carrier gas, the inlet temperature was 250 °C, and the column box was held at an initial temperature of 130 °C for 1 min, followed by heating from 130 °C to 160 °C at a rate of 10 °C·min^−1^, and held at the final temperature for 1.5 min. The sample solution (0.2 µL) was injected using a 1:15 split ratio. The thermogravimetric analysis (TGA) measurements of the sample were carried out using a thermogravimetric analyzer (TG209F3, NETZSCH-Geraetebau GmbH, Selb, Germany) in an oxygen atmosphere (20 mL·min^−1^) with nitrogen as a protective gas. The sample (2–5 mg) was heated from room temperature to 700 °C at a ramp rate of 5 °C·min^−1^.

### 2.5. Benzyl Alcohol Oxidation Test

The photocatalytic oxidation of benzyl alcohol experiment was carried out in a 50 mL three-necked quartz flask containing 10 mg of catalyst, 10 mmol of benzyl alcohol, and 2 mL of deionized water. A total of 1 mL of H_2_O_2_ (30%) was added to the reaction system through a continuous injection method. A 300 W xenon lamp (PLS-SXE300+, Perfectlight Technology Co., Ltd., Beijing, China) was used as a simulated sunlight and the reaction solution was vigorously stirred at room temperature. At the end of the reaction, the reaction solution was dewatered via toluene extraction and anhydrous magnesium sulphate, and the samples were analyzed using gas chromatography with nitrobenzene as an internal standard.

### 2.6. Photoelectrochemical Performance Tests

Photoelectrochemical performance tests were performed on an electrochemical workstation (CHI-660E, CH Instruments Inc., Shanghai, China) equipped with a standard three-electrode system. Typically, 5.0 mg of catalyst was dispersed in 1.0 mL of isopropanol/water mixture (3:1, *v*/*v*) and 15 μL of Nafion, and then dropped onto an ITO glass plate as the working electrode. A platinum sheet and a saturated Ag/AgCl electrode were used as the counter electrode and reference electrode, respectively, and 0.1 M Na_2_SO_4_ solution was used as the electrolyte. The photocurrent density was measured under 300 W xenon lamp irradiation with a light on/off period of 10 s. The photocurrent response curve (i-t curve) of the material was collected. The electrochemical impedance test was performed using the same sample preparation method with only a change in impedance solution at 10^5^ Hz for the high frequency and 1.0 Hz for the low frequency. The electrochemical impedance spectroscopy measurement was carried out to choose a mixed solution of 0.1 M KCl and 5.0 mM K_3_[Fe(CN)_6_]/K_4_[Fe(CN)_6_] as the electrolyte. The LSV curves were scanned over a range of 0.1 V to 1.0 V at a scanning rate of 0.1 V·s^−1^. For the LSV scan, 20 mg of the sample was dispersed in 480 μL ethanol and 20 μL of Nafion and then dripped onto one end of a 1 × 2 cm^2^ carbon paper as the working electrode. The electrolyte was an aqueous solution of Na_2_SO_4_ (0.1 M) and benzyl alcohol (0.1 M).

## 3. Results

**Figure 1:**
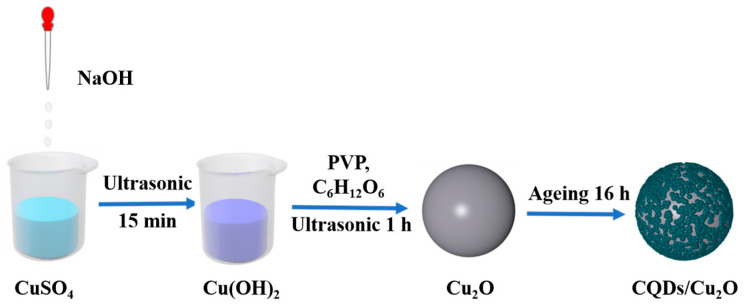
Schematic illustration of the synthesis process of CQDs/Cu_2_O.

**Figure 2:**
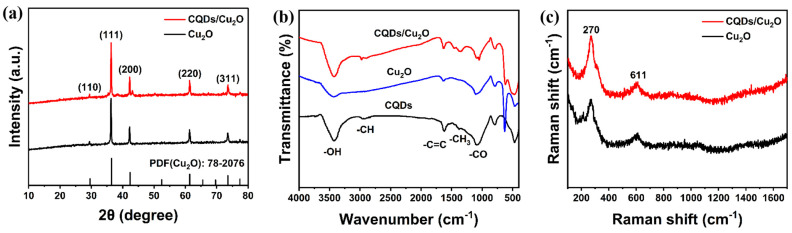
(**a**) XRD patterns of Cu_2_O and CQDs/Cu_2_O; (**b**) FT-IR spectra of CQDs, Cu_2_O and CQDs/Cu_2_O; (**c**) Raman spectra of Cu_2_O and CQDs/Cu_2_O.

The synthesis strategy of CQDs/Cu_2_O is shown in Figure 1. The X-ray diffraction patterns of Cu_2_O and CQDs/Cu_2_O are shown in Figure 2a. The 2θ peaks at 29.58°, 36.44°, 42.33°, 61.41°, and 73.56° for CQDs/Cu_2_O and pure Cu_2_O correspond to the cubic crystalline phases of Cu_2_O (PDF#78-2076) at (110), (111), (200), (220) and (311) crystal planes [26]. The XRD spectra of the composites clearly show the distinctive diffraction peaks of Cu_2_O, suggesting that the crystalline structure of Cu_2_O remains unaltered after its composite formation with CQDs. However, no significant carbon peaks are found due to the low loading quantity of CQDs. The surface functional groups of the prepared sample are analyzed via IR spectroscopy (Figure 2b). The characteristic functional groups observed on the CQDs surface are as follows: the -OH bond at 3425 cm^−1^, the -CH bond at 2918 cm^−1^, and the -C=C, -CH_3_, and -CO bonds at the positions of around 1619 cm^−1^, 1378 cm^−1^, and 1080 cm^−1^, respectively [27,28]. The peak at 629 cm^−1^ is ascribed to the stretching vibration of the Cu-O bond of Cu_2_O [29]. The successful combination of CQDs and Cu_2_O was verified. Figure 2c shows the Raman spectra of the prepared samples; the characteristic peak at 270 cm^−1^ corresponds to the bending vibration of the Cu-O bond in the Cu_2_O phase, and 611 cm^−1^ corresponds to the stretching vibration of the Cu-O bond [30]. The D-band (around 1350 cm^−1^) and G-band (around 1590 cm^−1^) signals from carbon quantum dots were not observed in the Raman spectra of the CQDs/Cu_2_O [31] due to the low concentration of CQDs in the composite. This result is in agreement with the results of the XRD tests.

**Figure 3:**
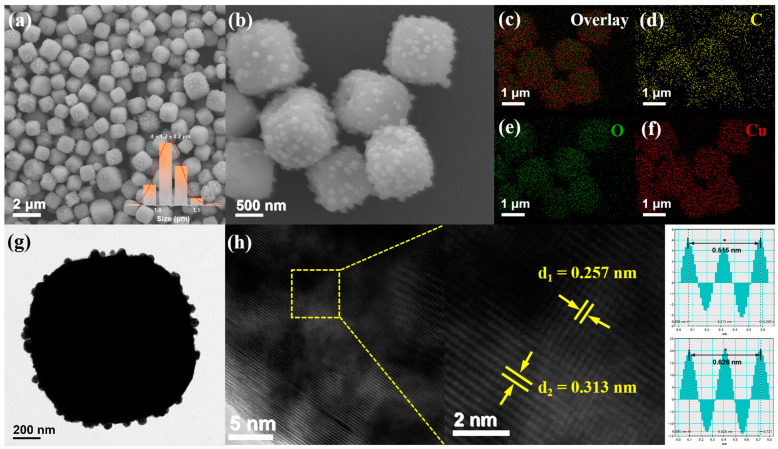
(**a**,**b**) SEM images of CQDs/Cu_2_O; (**c**) overlay image of SEM and elemental mapping data; (**d**–**f**) EDS elemental mapping profiles of CQDs/Cu_2_O with C (yellow), O (green) and Cu (red) distribution; (**g**) TEM image of CQDs/Cu_2_O sample; (**h**) HRTEM image of CQDs/Cu_2_O.

The scanning electron microscopy (SEM) and transmission electron microscopy (TEM) images of the composite (Figure 3) show that the CQDs/Cu_2_O composite has spherical morphology with protrusions on the surface with an average diameter of ca. 1.2 μm after aging for 16 h. The unique protruding structure of the CQDs/Cu_2_O composite allows light to be observed in multiple reflections between the big particle surface and small protruding particles, which can make greater use of solar energy and thus enhance its photocatalytic activity as compared to the smooth surface morphology of CQDs/Cu_2_O obtained by aging for 1 h [28]. The clear interface of CQDs successfully composited with Cu_2_O can also be observed in the high-resolution TEM (HRTEM) map (Figure 3h). The lattice spacing of 0.257 nm and 0.313 nm corresponds to the (100) crystalline plane of graphitic carbon [27,32] and the (110) crystalline plane of Cu_2_O, respectively [21,33]. The distribution of the elements of the EDS (Figure 3c–f) shows that C, O, and Cu elements are present in the composite with uniform distribution, indicating that the CQDs are successfully loaded on the surface of the Cu_2_O particle.

The results of the photocatalytic selective oxidation performance of benzyl alcohol by CQDs/Cu_2_O at room temperature are shown in Table 1. Entry 5 shows that the benzyl alcohol oxidation reaction is difficult to occur in the absence of any catalyst using H_2_O_2_ as a mild oxidant due to the inherent energy barrier of the reaction itself. Entry 1 shows that the photocatalytic oxidation of benzyl alcohol by CQDs/Cu_2_O at room temperature for 6 h shows a significant increase in conversion (35%) and exhibits a high selectivity for benzaldehyde (90%); however, the pure Cu_2_O only exhibits insignificant reactivity. In our experiment, we fixed the reaction time at 6 h due to slow kinetics in room temperature. This shows the important role of carbon quantum dots in the designed composites (CQDs/Cu_2_O). Firstly, the abundant oxygen-containing functional groups (e.g., hydroxyl groups, etc.) on the carbon quantum dots provide more activation sites and promote the interaction between the reaction components; secondly, the conjugated structure of the carbon dots promotes the adsorption of the substrate on the catalyst surface by the π–π interactions of the benzene ring [34]; and lastly, the introduction of the carbon quantum dots improves the energy band distribution of the Cu_2_O and facilitates the separation and movement of the carriers [35]. Entries 1–4 demonstrate that light is an important driving force for benzyl alcohol oxidation. Meanwhile, we also compared the photocatalytic performance of TiO_2_, a conventional photocatalyst (Entry 6), but its inherent wide bandgap and susceptibility to agglomeration both limit its ability to effectively utilize the visible light, resulting in low activity for benzyl alcohol oxidation [36].

**Figure 4:**
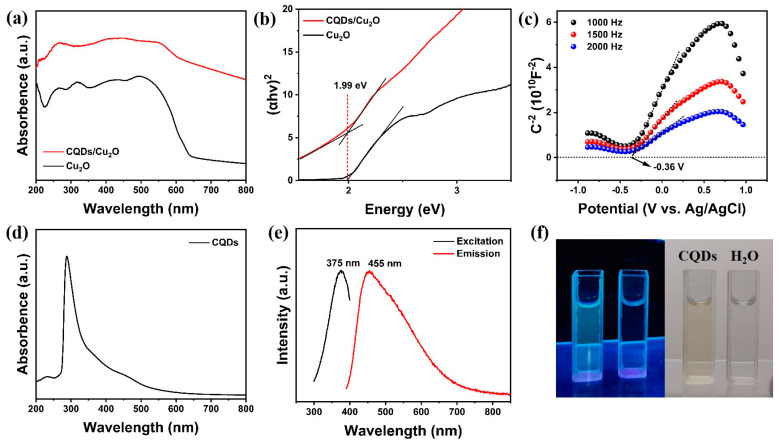
(**a**) DRS results of Cu_2_O and CQDs/Cu_2_O; (**b**) the estimated band gap energy (Eg) of Cu_2_O and CQDs/Cu_2_O calculated using the Kubelka–Munk function transformed from the DRS results; (**c**) Mott–Schottky curve of Cu_2_O; (**d**) UV spectrum and (**e**) PL spectra of CQDs solution; (**f**) CQDs optical images illuminated under UV light (left; 365 nm) and white (right; daylight lamp).

The light-harvesting capabilities of Cu_2_O and CQDs/Cu_2_O photocatalysts were evaluated through UV-Vis absorption spectra. As shown in Figure 4a, the absorption edge of CQDs/Cu_2_O shows an obvious shift in the visible region compared to Cu_2_O. In addition, CQDs/Cu_2_O can absorb more light in the 600–800 nm range, indicating the possibility of photocatalytic design of the complexes in the NIR region. The CQDs/Cu_2_O displays observably increased light-harvesting capabilities in the visible-light range, which may be attributed to the upconversion photoluminescence of CQDs on the Cu_2_O particles; this result is in agreement with the FT-IR and SEM observations. Figure 4d shows two absorption peaks of CQDs in the range of 233 nm and 270–400 nm [37]. The weak band at 233 nm corresponds to the π–π* electron excitation of the C=C bond, while the typical broad absorption region at 270–400 nm belongs to the n–π* electron transition of the C=O bond [38]. Based on the Kubelka–Munk function obtained from the conversion of the DRS results (Figure 4b), the optical bandgap value of CQDs/Cu_2_O amounts to 2.99 eV, which is the same as pristine Cu_2_O [39]. The Mott–Schottky plots (Figure 4c) show a positive slope that reflects that Cu_2_O belongs to the n-type semiconductor. Based on the intercept of the *x*-axis, the flat band (Efb) position was deduced to be about −0.36 eV. Therefore, the CB and VB positions of Cu_2_O are at −0.16 V and 1.82 V, respectively. The fluorescence emission characteristics of the CQDs are shown in Figure 4e–f, which shows a broader luminescence peak at about 455 nm upon excitation at 375 nm. A solution of the CQDs at 365 nm UV lamp shows yellow-green fluorescence. According to previous reports, the upconversion photoluminescence property of carbon quantum dots also plays a considerable role in the reaction [28]. It absorbs long-wavelength light and then emits short-wavelength light through upconversion, and also excites Cu_2_O to form electron–hole pairs, which is conducive to an enhancement in the photocatalytic oxidation activity of benzyl alcohol using the CQDs/Cu_2_O complex [35,40,41].

**Figure 5:**
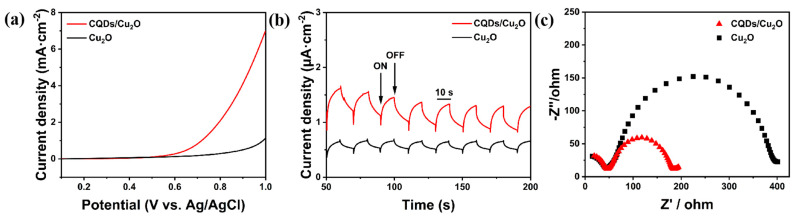
(**a**) LSV curves; (**b**) transient photocurrent response; (**c**) EIS Nyquist plots of Cu_2_O and CQDs/Cu_2_O.

The photoelectrochemical activity of the synthesized sample was investigated using linear scanning voltammetry (LSV) test. From the obtained LSV curves (Figure 5a), it can be seen that the Cu_2_O loaded with CQDs has a lower onset potential and significantly improves the electrical response as compared to the pristine Cu_2_O, suggesting that the CQDs/Cu_2_O catalytic reaction is more likely to occur. The current density of CQDs/Cu_2_O reached 4.11 mA·cm^−2^, which was almost 8.3 times higher than that of pristine Cu_2_O, at 0.9 V vs. Ag/AgCl. This is mainly due to the addition of CQDs, which has effectively increased the charge transfer rate. The charge transfer behavior in the composites is investigated by measuring the transient photocurrent response (Figure 5b) and electrochemical impedance spectroscopy (EIS) (Figure 5c). The composite material shows a better photoelectric response in visible light compared to pristine Cu_2_O, which indicates more efficient separation and transfer of photogenerated charges [42]. This can be attributed to the introduction of CQDs, resulting in photogenerated electrons with sufficient energy to be captured by Cu_2_O. In the electrochemical impedance plot, the arc radius of CDs/Cu_2_O is smaller, indicating lower resistance and faster interfacial charge transfer [43]. These results fully reflect the fast interfacial charge transfer in the composites, which improves the catalytic performance of the CQDs/Cu_2_O.

**Figure 6:**
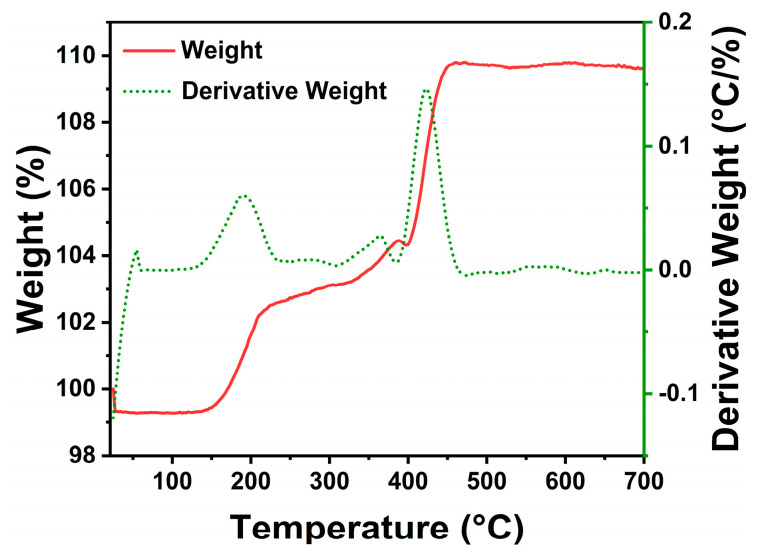
Thermogravimetric analysis (TGA) of the CQDs/Cu_2_O sample.

Figure 6 illustrates the thermogravimetric analysis of the CQDs/Cu_2_O sample. The first stage of mass loss (0.7%) occurs in the range of 25–125 °C, corresponding to the evaporation of adsorbed water molecules in the sample [44]. The weight gain (10.5%) observed in the temperature range of 125–460 °C is attributed to the gradual oxidation of Cu_2_O to black CuO in the presence of O_2_ under elevated temperatures, with the maximum reaction rate occurring at 422 °C. The stage between 230 and 400 °C corresponds to the decomposition of oxygen-functional groups in CQDs [45,46]. Through calculations, the estimated content of CQDs and Cu_2_O in the composite material is approximately 5.4% and 93.9%, respectively. This aligns closely with the results obtained from SEM testing. These results indicate the high thermal stability of CQDs/Cu_2_O composite for catalytic purposes and especially for the oxidation of benzyl alcohol at the desired temperature.

**Figure 7:**
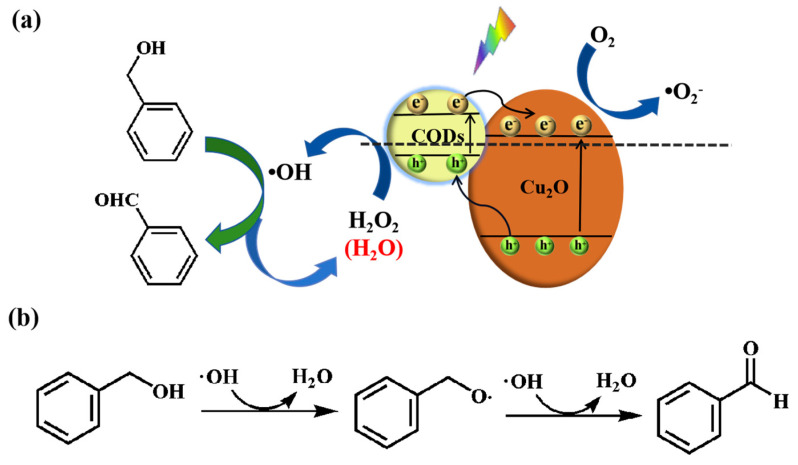
(**a**,**b**) Schematic mechanism for the catalytic oxidation of benzyl alcohol by CQDs/Cu_2_O.

Based on the above experimental results and published reports, a possible catalytic mechanism for CQDs/Cu_2_O has been proposed (Figure 7). Firstly, CQDs/Cu_2_O generate photogenerated electron–hole pairs under light stimulation, and the rapid charge transfer at the interface of the two components due to the heterogeneous structure formed between CQDs and Cu_2_O alleviates the problem of carrier recombination [47]. Meanwhile, the upconversion fluorescence property of CQDs and the protruding nanostructures of Cu_2_O particles with excellent light-reflecting ability enable the composite to serve as an efficient and stable visible-light-sensitive photocatalyst. Then, the mild oxidizing agent H_2_O_2_ combines with photogenerated holes (h^+^) to react and decompose to generate hydroxyl radicals (·OH), an active species with strong oxidizing properties [17]. Next, the free radical of ·OH causes the dehydrogenation of benzyl alcohol adsorbed on the catalyst to produce the ·C intermediate, and this intermediate is subsequently further oxidized by ·OH to produce benzaldehyde. Notably, in the case of light irradiation, CQDs can act as powerful electron donors for the first step of benzyl alcohol oxidation (benzaldehyde) protected by photo-induced electron transfer, and the product is prevented from being over-oxidized by the reducing environment provided by the photogenerated electrons (e^−^), and thus the reaction can obtain a high benzaldehyde selectivity (90%).

## 4. Conclusions

In summary, CQDs/Cu_2_O composites were synthesized using a simple one-step sonication method for the light-stimulated selective oxidation of benzyl alcohol at room temperature. CQDs were uniformly distributed on the surface of Cu_2_O and formed a heterojunction structure, which improved the charge carrier separation efficiency and provided more sites for benzyl alcohol adsorption. In addition, the protruding structure of Cu_2_O and the upconversion fluorescence of the CQDs allowed for multiple reflections of light and improved the efficiency of the composite material in utilizing sunlight energy. Photogenerated vacancies play a major role in benzyl alcohol oxidation, and h^+^ allows the decomposition of H_2_O_2_ to generate hydroxyl radicals with strong oxidative potential. Meanwhile, CQDs act as electron donors and provide a reduced environment for the protection of benzaldehyde from over-oxidation. Finally, it was illustrated that the incorporation of carbon quantum dots has broadened the light-absorbing properties of the materials, which has considerable positive effects on the activity, selectivity, and stability of the benzyl alcohol oxidation process. This work has achieved significant positive results in the effective design of photocatalysts and in solving the environmental pollution and energy consumption problems faced by traditional organic production.

## Figures and Tables

**Table 1:** Photocatalytic activity for the selective oxidation of benzylic alcohols over CQDs/Cu_2_O under visible-light irradiation.

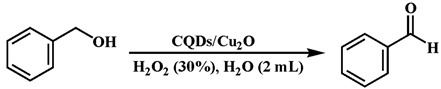					
Entry	Catalyst	Time (h)	T (°C)	Conversion (%)	Selectivity (%)
1	CQDs/Cu_2_O (hv)	6	r.t.	35	90
2	CQDs/Cu_2_O	6	r.t.	2	100
3	Cu_2_O (hv)	6	r.t.	10	100
4	Cu_2_O	6	r.t.	-	-
5	None	6	r.t.	-	-
6	TiO_2_ (hv)	6	r.t.	13	100
7	g-C_3_H_4_ (hv)	6	r.t.	12	100
8	CQDs (hv)	6	r.t.	18	91

Reaction condition: benzyl alcohol (10 mmol), photocatalyst (10 mg), H_2_O_2_ (1 mL, 30%), H_2_O (2 mL), temperature (25 °C), irradiation time (6 h).

## Data Availability

Data are contained within this article.
